# AI Survival Prediction Modeling: The Importance of Considering Treatments and Changes in Health Status over Time

**DOI:** 10.3390/cancers16203527

**Published:** 2024-10-18

**Authors:** Nabil Adam, Robert Wieder

**Affiliations:** 1 Phalcon, LLC, Manhasset, NY 11030, USA; n.adam@phalconllc.com; 2 Newark Campus, Rutgers University, Newark, NJ 07102, USA; 3 Rutgers New Jersey Medical School, Newark, NJ 07103, USA; 4 Rutgers Cancer Institute of New Jersey, Newark, NJ 07103, USA

**Keywords:** deep learning, survival models, breast cancer, SEER-Medicare linked dataset

## Abstract

**Simple Summary:**

Predictions of survival in patients with localized breast cancer base their models on data from the time the patients are diagnosed. These survival curves have an inherent inaccuracy because they do not take into consideration events that occur after initial diagnosis. We used eep learning, a type of artificial intelligence, to model the survival of Medicare patients with stage I–III breast cancer from the SEER-Medicare dataset from 1991 to 2016. In addition to considering patient and cancer variables from the time of diagnosis, we included variables that occurred later, including treatment, adverse events, other medical conditions, and progressive age of the patient. Our predictions improved significantly, with the inaccuracy rate dropping from around 30% to less than 10% when the time-varying data were added to the time-fixed data. We also developed our models to generate individual patient predicted survival based on their unique circumstances made up of the patient, cancer, treatment, and treatment-related adverse events that occurred over time. This approach will be a powerful tool that can advise oncology caregivers and patients on the factors that impact their predicted survival.

**Abstract:**

Background and objectives: Deep learning (DL)-based models for predicting the survival of patients with local stages of breast cancer only use time-fixed covariates, i.e., patient and cancer data at the time of diagnosis. These predictions are inherently error-prone because they do not consider time-varying events that occur after initial diagnosis. Our objective is to improve the predictive modeling of survival of patients with localized breast cancer to consider both time-fixed and time-varying events; thus, we take into account the progression of a patient’s health status over time. Methods: We extended four DL-based predictive survival models (DeepSurv, DeepHit, Nnet-survival, and Cox-Time) that deal with right-censored time-to-event data to consider not only a patient’s time-fixed covariates (patient and cancer data at diagnosis) but also a patient’s time-varying covariates (e.g., treatments, comorbidities, progressive age, frailty index, adverse events from treatment). We utilized, as our study data, the SEER-Medicare linked dataset from 1991 to 2016 to study a population of women diagnosed with stage I–III breast cancer (BC) enrolled in Medicare at 65 years or older as qualified by age. We delineated time-fixed variables recorded at the time of diagnosis, including age, race, marital status, breast cancer stage, tumor grade, laterality, estrogen receptor (ER), progesterone receptor (PR), and human epidermal receptor 2 (HER2) status, and comorbidity index. We analyzed six distinct prognostic categories, cancer stages I–III BC, and each stage’s ER/PR+ or ER/PR− status. At each visit, we delineated the time-varying covariates of administered treatments, induced adverse events, comorbidity index, and age. We predicted the survival of three hypothetical patients to demonstrate the model’s utility. Main Outcomes and Measures: The primary outcomes of the modeling were the measures of the model’s prediction error, as measured by the concordance index, the most commonly applied evaluation metric in survival analysis, and the integrated Brier score, a metric of the model’s discrimination and calibration. Results: The proposed extended patients’ covariates that include both time-fixed and time-varying covariates significantly improved the deep learning models’ prediction error and the discrimination and calibration of a model’s estimates. The prediction of the four DL models using time-fixed covariates in six different prognostic categories all resulted in approximately a 30% error in all six categories. When applying the proposed extension to include time-varying covariates, the accuracy of all four predictive models improved significantly, with the error decreasing to approximately 10%. The models’ predictive accuracy was independent of the differing published survival predictions from time-fixed covariates in the six prognostic categories. We demonstrate the utility of the model in three hypothetical patients with unique patient, cancer, and treatment variables. The model predicted survival based on the patient’s individual time-fixed and time-varying features, which varied considerably from Social Security age-based, and stage and race-based breast cancer survival predictions. Conclusions: The predictive modeling of the survival of patients with early-stage breast cancer using DL models has a prediction error of around 30% when considering only time-fixed covariates at the time of diagnosis and decreases to values under 10% when time-varying covariates are added as input to the models, regardless of the prognostic category of the patient groups. These models can be used to predict individual patients’ survival probabilities based on their unique repertoire of time-fixed and time-varying features. They will provide guidance for patients and their caregivers to assist in decision making.

## 1. Introduction

Many factors affect long-term survival from localized breast cancer (BC) in women. Covariates that impact predictive survival modeling can be both time-fixed and time-varying, and each have been incorporated in generating the predicted survival of BC patient populations. Time-to-event modeling aims to predict the patients’ survival function. Factors that correlate with survival from localized breast cancer are covariates that characterize the patients and their cancer at the time of diagnosis. These factors, referred to as time-fixed covariates, include the cancer stage (I–III), laterality, tumor size (T), number of lymph nodes (N), estrogen receptor (ER), progesterone receptor (PR), and human epidermal growth factor receptor 2 (Her2) status, tumor grade, Ki67 staining, lymphovascular invasion, perineural invasion, androgen receptor (AR) status, molecular signature and recurrence score, stem cell frequency, the presence of circulating tumor cells and bone marrow micrometastases, patient race, location of residence, age, menopausal status, relationship status, health, and comorbidity status [1,2,3,4,5,6,7,8,9,10,11,12,13,14,15,16,17,18,19,20,21,22].

However, much happens during patients’ lives after the initial diagnosis that affects their survival. These factors are referred to as time-varying covariates, and their impact on survival is less well-characterized, more complex, and often as important as the impact of time-fixed covariates. Time-varying covariates that affect survival include the administration of neoadjuvant therapy, timing and type of initial surgery, the timing of initiation, amount, type, and completion of adjuvant chemotherapy, biotherapy, hormone therapy, radiotherapy, or prolonged hormone or Her2 blockade [14,19,23,24]. Multiple life events that occur at irregularly variable periods have been implicated in decreased survival [25]. They include changes in health status, adverse events from treatment, comorbidities, post adjuvant reconstruction [26] or other non-cancer surgery, angiogenesis and wound healing, hypercoagulable states, persistence of bone marrow disseminated tumor cells [27], aging, estrogen deprivation, infection, inflammation, exposure to toxic chemicals, sedentary lifestyle, weight gain, obesity, smoking, alcohol consumption [28,29,30], anxiety, stress, and depression [25]. These events shorten survival independently or by abrogating the microenvironment’s ability to suppress the awakening of dormant micrometastases [25].

Given the proven impact of both time-fixed and time-varying covariates on predicted survival in population-averaged models, we hypothesized that taking them both into consideration would improve the accuracy of predictive modeling. We focused our study on prediction models that use time-to-event data, including the time a patient is observed for the event of interest, death in our case, and whether they experienced it before the study follow-up ended. Some patients are said to be right-censored, i.e., are lost to the study before experiencing the event and before the study follow-up ends or did not experience the event before the study follow-up ends. Over the past five decades, statistical methods for analyzing time-to-event data, especially right-censored survival data, have been developed. Unlike traditional prediction models, survival prediction models account for censoring; ignoring the censoring leads to biased and inefficient predictions [31,32]. Additional shortcomings of population-based predictions are that they provide highly inadequate predictions of individual patient outcomes due to the virtually unlimited combination of both time-fixed and time-varying features of each patient that impact survival.

Parametric survival models assume a specific distribution, e.g., Weibull, for the survival times. These models, however, lead to biased estimates when the assumed survival time distribution is violated [33]. The semiparametric Cox Proportional Hazards (CPH) regression model [34] is the most common time-to-event analysis approach in the medical literature. Most survival models, including the CPH model, are designed for data with continuous failure time distributions. In real life, patient follow-up visits occur on a given day with irregular gaps between two consecutive visits. Applying standard continuous-time models on discrete-time data without adequate adjustments can lead to biased estimators [35,36]. Further, the CPH model’s proportionality hazard assumption, i.e., the effect of each patient covariate is the same at all values of the follow-up time, is unrealistic for most clinical situations [37].

As a nonlinear extension to CPH, Faraggi–Simon’s network [38] was an early attempt to extend CPH with a neural network. Since the state of development of neural networks was not as advanced as it is today, the results did not show improvement beyond the linear CPH model. Given the modern era of high-performance computing and available datasets of hundreds of thousands of patients and hundreds of millions of patient records, deep learning (DL) models, an artificial intelligence (AI) subfield, are increasingly common approaches to developing survival prediction using time-to-event data. AI-based survival models, specifically DL models, capture the complex nonlinear relationships among the patient’s characteristics, cancer characteristics, treatments, adverse events, comorbidity, etc. These models help achieve precision medicine, providing guidance for treatment that is personally tailored to individual patients with stage I–III BC.

## 2. Methods

### 2.1. Deep Learning Predictive Modeling

#### 2.1.1. Discrete Time-to-Event Data

**DeepSurv**. The first successful attempt to extend the Cox regression model with neural networks (NNs) was proposed in [39], where the patient’s covariates are input to the network, and the single node out of the network uses a linear activation function to estimate the log-risk function in the Cox model. Their results demonstrated that NNs were able to outperform classical Cox models.

**DeepHit**. Lee et al. [40] were the first to apply NNs to the discrete-time likelihood for right-censored time-to-event data [41]. Their DL-based model, DeepHit, treated survival time as discrete and the time horizon as finite. The model makes no assumptions about the underlying stochastic process by directly learning the joint distribution of survival times and events and allowing for the possibility that the relationship between covariates and risk(s) changes over time. Their results showed that DeepHit outperformed previous models.

**Nnet-survival**. In [37], a discrete-time survival model, Nnet-survival, was proposed. Given input data of *n* patients, each with covariate vector $x_{i}$, we can fit the model by minimizing the loss given by the mean negative log-likelihood. Their result showed “good” discrimination and calibration performance with simulated and real data.

**Cox-Time**. To overcome the proportionality assumption of the Cox model, Kvamme et al.’s [41] proposal was to consider time as a regular covariate and modify the relative risk function to have it dependent on time, resulting in $h\left(t|x\right)=h_{0}\left(t\right)e^{g\left(t,x\right)}$. Thus, $g\left(t,x\right)$ model interactions between time and the other covariates are considered. This model, Cox-Time, although no longer a proportional hazard model, is still a relative risk model with the same partial likelihood as the Cox model with the following loss function, $\;loss=\frac{1}{n}\;\sum_{i:D_{i}=1}\log\sum_{j\epsilon \tilde{R_{i}}}e^{\left[g\left(T_{i},x_{j}\right)-g\left(T_{i},x_{i}\right)\right]}$. The model is trained on continuous-time data but produces discrete-time predictions.

To summarize, DeepSurv is limited by the proportionality assumption of the CPH model, whereas DeepHit, Nnet-Survival, and Cox-Time are not restricted by the proportionality assumption. DeepSurv is designed for data with continuous failure time distributions; Cox-Time, on the other hand, is trained on continuous-time data but produces discrete-time predictions. Nnet-Survival is a discrete-time model where the baseline hazard rate and the effect of the input data on hazard probability can vary with follow-up time.

#### 2.1.2. Time-Varying Covariates: Proposed Extension

A patient has two sets of covariates: time-fixed covariates (e.g., age at diagnosis) and time-varying covariates (e.g., current age, current comorbidity index, treatments administered at this visit and earlier visits, and adverse events). A patient’s survival status is recorded at each visit while the patient is at risk, i.e., has not yet experienced the event (death in our case) of interest.

The above methods, while dealing with discrete-time data, assume that the $\;x_{i}$ covariates of a patient, i, are time-fixed. A realistic prediction of the patient’s survival needs to consider not only the patient’s covariates at the time of diagnosis but also the administered treatments, induced adverse events, comorbidity index, and the age at each visit.

To achieve this objective, we extended a patient covariate vector, $\;x_{i}$, to include not only time-fixed covariates but also covariates that summarize the patient’s history from previous visits. Specifically, for a given treatment, $\;{TR}_{j}\;for\;j=1,\;2,\ldots ,\;46$, ${TR}_{ij}$ is a tally of the number of times ${TR}_{j}$ was administered to this patient, i, from the time of the diagnosis to the time of death/end of the study. Similarly, for a given adverse event induced, $\;{AE}_{k}\;for\;k=1,\;2,\ldots ,\;18$, ${AE}_{ik}$ is a tally of the number of times the patient, i, experienced ${AE}_{k}$, from the time of the diagnosis to the time of death/end of the study. We divide the age of a patient into 6 bins: $bin_{1}\;$≤ 65, 65 < $bin_{2}\;$ ≤ 70, 70 < $bin_{3}\;$ ≤ 75, 75 < $bin_{4}\;$ ≤ 80, 80 < $bin_{5}\;$ ≤ 85, $bin_{6}\;$ > 85. ${AGE}_{ib}$ is a tally of the number of times the age of patient, i, falls within the $bin_{b}\;for\;b=1,\;2,\;\ldots ,\;6$, from the time of the diagnosis to the time of death/end of the study. We handle a patient comorbidity index similar to a patient’s age. We divide the comorbidity index of a patient into 6 bins: $bin_{1}\;$ ≤ 2, 2 < $bin_{2}\;$ ≤ 4, 4 < $bin_{3}\;$ ≤ 6, 6 < $bin_{4}\;$ ≤ 8, 8 < $bin_{5}\;$ ≤ 10, $bin_{6}\;$ > 10. $COMB_{ib}$ is a tally of the number of times the comorbidity index of the patient, i, falls within the 6 comorbidity bins: 1, 2, …, 6 from the time of the diagnosis to the time of death/end of the study.

### 2.2. Experiments

We compared the performance of the four predictive models discussed above when using the patients’ time-fixed covariates versus when using our proposed extended patients’ covariate vectors to include time-fixed covariates and covariates that summarize the patient’s history from previous visits.

#### 2.2.1. Study Data: SEER-Medicare Linked Dataset

We utilized the SEER-Medicare linked dataset, which provides information on cancer care and outcomes of Medicare beneficiaries with cancer [42]. Medicare data have a patient Entitlement and Diagnosis Summary File, a person-level file that provides SEER demographic and clinical information for up to 10 primary cancer diagnoses, treatments, and mortality. Medicare files capture the fee-for-service claims from hospitals, outpatient facilities, National Claims History, hospice care, home health agencies, and Part D Prescription Drug Event claims. The CCflag file includes, for every patient, the date the patient was diagnosed with one of twenty-two chronic conditions. We used these data to compute a time-varying patient’s comorbidity index. There were 883,053 BC patients during 1991–2016. We fused the data in the various files based on the Observational Medical Outcomes Partnership (OMOP) common data model. These data within those disparate files are transformed into a common format (data model) and a common representation (terminologies, vocabularies, coding schemes).

#### 2.2.2. Cohort Selection

In our study, we included women with the diagnosis of stage I–III breast cancer who have not had any other malignancy history except non-melanoma skin and eyelid cancer, as a standard in NCI clinical trials [43], included all comorbidities recorded at every visit from the date of diagnosis, averaged over the course of all the treatments assessed for each patient, all prior treatments, delineated age, race, marital status, breast cancer stage, tumor grade, laterality, and ER status, PR, and HER2 status. We only included patients whose age at enrollment was 65 or older and qualified by age, not disability. The enrolled population was, therefore, skewed by age and only represented an elderly subset of the breast cancer patient population. We included patients who were enrolled in both Parts A and B of Medicare with no HMO enrollment from 1 month prior to diagnosis through 20 years following diagnosis, hospice, or death to ensure that subjects were continuously enrolled in the proper parts of Medicare during the study period.

For our analysis, we divided the patient population into groups of patients with ER/PR + and − cancers, which are diseases with different genetic, behavioral, and survival characteristics [9], and patients with stages I, II, and III cancers, which have vastly different prognoses from each other [16]. Treatment affects patients with these different classifications differently according to guidelines developed based on clinical investigations [19,44,45].

#### 2.2.3. Data Cleaning, Standardization, Encoding, and Embedding

We removed duplicate records and included alid data, e.g., ICD and HCPCS codes, dates, etc. We performed data transformation and standardization. We categorized treatments into 46 mechanistic categories and adverse events into 18 categories reported in the BC treatment literature. We applied embeddings, which resulted in the efficient computation and discovery of complex patterns, reduced overfitting, and captured the underlying structure of the data to better generalize new, unseen data [46].

#### 2.2.4. Performance Metrics

The concordance index (C-index) is the most commonly applied discriminative evaluation metric in survival analysis [41]. The cause-specific time-dependent C-index, which explicitly accounts for censoring, estimates the model’s prediction error [47]. We also measured the model’s performance using the integrated Brier score (IBS) [48,49,50], a discrimination metric, and a calibration metric of a model’s estimates.

#### 2.2.5. Model Hyperparameters

**Figure 1:**
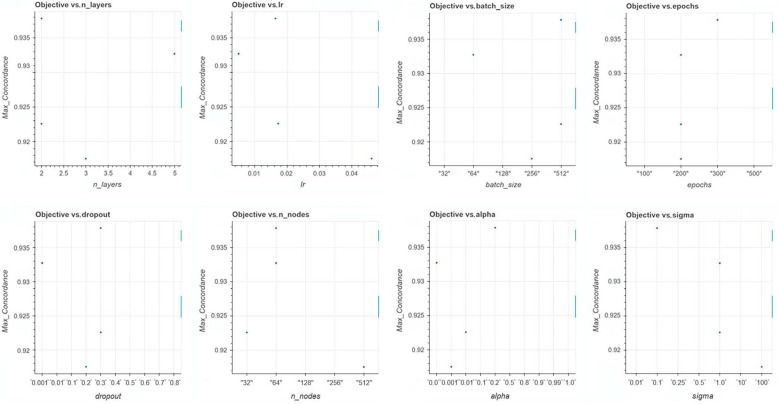
The C-index vs. the model hyperparameters: n-layers, Ir, batch size, epochs, dropout, n_nodes, alpha, sigma.

These neural network parameters are fixed by design and not tuned by training; they should be optimized. We applied Amazon SageMaker Python SDK 2.232.2 (software development kit), an open-source library, to fine-tune the model by identifying optimal values of the network’s hyperparameters. We used a Bayesian optimization search scheme [51]. Table 1 includes the list of the hyperparameters and their ranges. Figure 1 depicts the correlation between the performance metric, time-dependent concordance, and each of the model’s hyperparameters for the case of stage I and ER+.

#### 2.2.6. Models Validation

We used the pycox package [41]. To validate our implementation, we applied the four models, DeepSurv, DeepHit, Nnet-survival, and Cox-Time, to the real datasets METABRIC [52] and SUPPORT [53]. The experiments were conducted using five-fold cross-validation. As shown in Table 2, our results confirm that “all methods perform quite similarly” [54] and are aligned with published results of each of these models using these datasets [54].

## 3. Results

The patient characteristics, including the number of entries and patients in the category, mean age, and comorbidity indices at the time of initial diagnosis, are presented in Table 3. The data concur with our prior observations with the SEER-Medicare file that ER− patients represent 17.3% of the patients. In the ER+ category, 59.7% of the patients had stage I cancer, and only 9.6% of patients had stage III cancer, while in the ER− group, only 42.8% of the patients had stage I disease, while 18.4% had stage III disease. These data show later-stage distributions in more aggressive ER− tumors than ER+ tumors [16].

The recurrence rates and survival of patients with ER+/PR+ cancers and ER−/PR− cancers vary with well-described characteristics [9,16]. To assess the degree of sensitivity of the model’s predictive accuracy and the model’s calibration to the ER/PR status of the patient population, we considered the following six scenarios of BC patients: scenario 1: stage I, ER/PR+; scenario 2: stage II, ER/PR+; scenario 3: stage III, ER/PR+; scenario 4: stage I, ER/PR−; scenario 5: stage II, ER/PR−; and Scenario 6: stage III, ER/PR−.

Table 4 demonstrates the predictive performance of the four models when considering only the time-fixed covariates versus when considering the time-fixed and the time-varying covariates. In terms of concordance, we observe the significant improvement of the model with the proposed extended patients’ covariates compared with that of the patients’ time-varying covariates. For example, the DeepSurv model’s prediction error is 4% when using the proposed extended patients’ covariates versus over 32% when using the patients’ time-fixed covariates.

Our results demonstrate that the performance of each of the models considered is relatively insensitive to the patient’s ER/PR status, both when considering only the patient’s covariates at diagnosis, or the patient’s time-varying covariates in addition to the patient’s covariate at diagnosis. The predictive capacity of the models improves significantly regardless of ER/PR status or stage when time-varying covariates are considered together with the time-fixed covariates.

**Figure 2:**
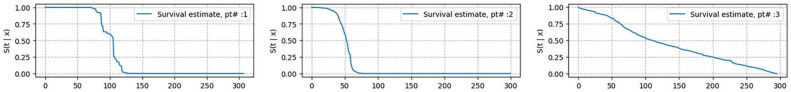
Predicted survival curves for three hypothetical patients. Details of data used to produce these hypothetical predicted survival curves are not permitted by NCI SEER-Medicare because they were derived from individual patient-identifying information.

To illustrate the practical application of the prediction models to clinical scenarios, we selected three hypothetical patients with unique individual patient, cancer treatment, adverse events features, and progressive age and comorbidity indices, and generated predicted survival curves based on their time-fixed and their time-varying variables (Figure 2). These data demonstrate the potential application of the model to individual patients with their unique characteristics to predict their own individual survival probabilities. We compared each of these hypothetical patient’s median predicted survival probability and the population-averaged predicted survival probability by age from Social Security tables [55], and stage and race [16]. Our data demonstrate that both sets of population-averaged predicted values are vastly different from the predicted median survival of each patient generated by the models. Our model-predicted survivals are influenced by the standard time-fixed variables of age, race, stage, hormone and Her2 status, tumor grade, as outlined in countless prior studies referenced above, as well as by the impact of treatments, treatment-associated adverse events, age and comorbidity progression with treatment, which collectively have positive and negative impacts on the relationship of the model-based survival and population-averaged survival.

### Model Interpretability

AI-based model understanding, an active area of research, helps provide insights into the models’ decision-making process. Examples of methods that attempt to break the “black-box” characterization of AI-based models are LIME (Local Interpretable Model-agnostic Explanations) [56], SHAP (SHapley Additive exPlanations) [57], and Captum, which is a state-of-the-art open-source, comprehensive library for deep learning PyTorch model explainability [58]. Limitations of these methods include computational complexity and instability, i.e., different runs may produce different explanations for the same instance.

In addition, interpreting the resulting covariate importance is a challenge, especially, as is the case in our environment, when the input covariates interact in a complex manner, resulting in computed attribution scores that do not capture the nonlinear dependencies between the network inputs and outputs.

## 4. Discussion

Our findings present a unique and compelling opportunity to improve the prediction performance of the four DL models that handle discrete-time distributions by extending the patients’ covariates vectors to include both time-fixed covariates and covariates that summarize the patient’s history from previous visits. The IBS can be viewed as the mean square error of prediction; lower values of the IBS indicate better predictive performance.

Our analyses using time-fixed covariates in six different prognostic categories all demonstrated an error rate ranging from approximately 28% to 37%. When we combined time-varying covariates that included treatments, adverse events, aging, and comorbidities, the accuracy of all four predictive models improved significantly, with error rates decreasing to 0.4–16%. Although published survival predictions derived from time-fixed covariates vary significantly between stage I, II, and III cancers and between ER/PR+ and ER/PR− cancers, all four of the predictive models demonstrated the same narrow range of inaccuracy when trained on time-fixed covariates and all improved relatively equally to highly accurate estimates when we incorporated time-varying covariates into the modeling.

We can hypothesize that these trends result from our proposed extension to combine multiple time-varying events in the modeling. Indeed, survival from localized breast cancer is the result of the cumulative effects of multiple covariates that have a collective impact on time to death. These time-varying events include wide-ranging treatment categories [14,19,23,24], adverse events from treatment [59,60,61], patient health and mental health events [62,63,64], progressive age, and frailty [65]. Studies generally investigate treatments individually or in combination on their impact on recurrence and survival, which are linked nonlinearly based on a number of factors [19,44,66,67]. Most of the time-varying events have an impact individually on recurrence and survival, factors that depend on the initial cancer stage and hormonal status [15,68]. However, the totality of these time-varying events and their nuanced impact on individual scenarios resulted in a global improvement of predictability nearing unity.

There are several potential limitations to this study. Our patient population consisted of Medicare-enrolled patients over 65 who qualified by age and not for other medical conditions. Their age range, therefore, is not representative of the general population, and their overall expected survival may be less than that of patients with stage I–III BC at a younger age. They may also not be necessarily representative of patients with private insurance coverage. Due to the enrollment criteria, patients may have been diagnosed with BC and potentially received treatment before enrollment in Medicare, potentially skewing the results based on Medicare claims. To generalizthe applicability of our conclusions of significant improvements in the accuracy of survival predictions by combining time-varying covariates with time-fixed covariates, we will recapitulate these approaches in the Medicaid datasets that are composed of younger patients more representative in age of the general population. The limitations of the older ICD-9 diagnosis codes and the lack of recurrence data will need to be addressed using additional datasets [69]. Nevertheless, the predictive modeling accuracies were highly concurrent among the four models in all disease scenarios, suggesting a similar efficacy when considering these additional confounding variables.

DL-based prediction models exhibit outstanding performance; predictive models for BC recurrence and survival often focus on limited covariates related to tumor, treatment, molecular, and clinical covariates. As part of a follow-up investigation, we plan to conduct an in-depth study that builds on our preliminary experimentation with Captum, where we applied the integrated gradient-based method. We plan to study Camptum’s performance when applying DeepLift, FeatureAblation, and ArchDetect methods. Future investigations will focus on molecular characteristics and gene expression characteristics of different cancers to incorporate their impact on predictive probabilities. Future investigations of these models will also be conducted using datasets that record recurrence as well to address additional time-to-event endpoints, including time-to-recurrence and recurrence-free survival.

## 5. Conclusions

Our data demonstrate that predicting the survival of stage I–III BC patients using only time-fixed variables suffers from a significant error rate of around 30%. However, adding time-varying covariates to the time-fixed covariates in predictive modeling using four DL models significantly decreases the error rate to around 10%, regardless of the prognostic category of the patient prognostic groups with widely differing predicted survival hazard curves based on time-fixed data. The application of these models of individual patients led to predicted survival probabilities that are vastly different and more accurate than population-averaged data based on time-fixed variables based on race, stage, patient overall health, and activity. This approach will have a significant impact on improving the faithfulness of survival estimates based on the unique variables of individual patients and can be applied as an adjunct tool in the clinical care of stage I–III BC patients.

## Figures and Tables

**Table 1:** Hyperparameters.

Hyperparameter	Type	Range
Batch size	Categorical	[32, 64, 128, 256, 512]
Epochs	Categorical	[100, 200, 300, 500]
Dropout rate	Categorical	[0.001, 0.01, 0.1, 0.2, 0.3, 0.4, 0.5, 0.6, 0.7, 0.8]
Number of layers	Integer values	[2, 5]
Number of nodes	Categorical	[32, 64, 128, 256, 512]
Alpha	Categorical	[0.0, 0.001, 0.1, 0.2, 0.5, 0.8, 0.9, 0.99, 1.0]
Sigma	Categorical	[0.01, 0.1, 0.25, 0.5, 1.0, 10, 100]
Learning rate	Continuous	[0.0001, 0.1]

**Table 2:** Validation of applied models.

Model	Time-Dependent Concordance				Integrated Brier Score			
	Support		Metabric		Support		Metabric	
	As Reported in the Literature	Our Results	Reported in the Literature	Our Results	Reported in the Literature	Our Results	Reported in the Literature	Our Results
Cox-Time	0.630	0.647	0.664	0.683	0.212	0.182	0.173	0.150
DeepHit	0.639	0.646	0.675	0.676	0.227	0.196	0.186	0.103
DeepSurv	0.615	0.630	0.640	0.710	0.213	0.231	0.175	0.136
Nnet-Survival (Logistic Hazard)	0.625	0.617	0.658	0.674	0.184	0.205	0.172	0.142

**Table 3:** Patient characteristics.

Stage	Number of Entries	Number of Patients	Age ± SD	Comorbidity Index ± SD	Number of Entries	Number of Patients	Age ± SD	Comorbidity Index ± SD
	ER/PR+				ER/PR−			
I	17,400,569	92,467	74.7 ± 6.8	2.9 ± 2.9	2,741,780	13,880	74.0 ± 6.7	2.8 ± 2.9
II	8,828,801	47,469	75.7 ± 7.5	2.9 ± 3.1	2,292,617	12,560	75.2 ± 7.5	2.6 ± 3.0
III	2,604,115	14,825	75.8 ± 7.6	2.5 ± 3.0	979,047	5966	75.7 ± 7.7	2.1 ± 2.9

**Table 4:** Time-dependent concordance and integrated Brier score.

Model	Time-Dependent Concordance		Integrated Brier Score		Time-Dependent Concordance		Integrated Brier Score	
	SM_Time-Fixed Patients’ Covariates ± SD	SM_Time-Fixed & Varying Patients’ Covariates ± SD	SM_Time-Fixed Patients’ Covariates ± SD	SM_Time-Fixed & Varying Patients’ Covariates ± SD	SM_Time-Fixed Patients’ Covariates ± SD	SM_Time-Fixed & Varying Patients’ Covariates ± SD	SM_Time-Fixed Patients’ Covariates ± SD	SM_Time-Fixed & Varying Patients’ Covariates ± SD
	ER/PR+				ER/PR−			
Stage I								
Cox-Time	0.679 ± 0.001	0.987 ± 0.001	0.112 ± 0.002	0.009 ± 0.003	0.690 ± 0.005	0.987 ± 0.002	0.120 ± 0.002	0.011 ± 0.001
DeepHit	0.667 ± 0.002	0.958 ± 0.001	0.110 ± 0.003	0.013 ± 0.001	0.671 ± 0.003	0.960 ± 0.003	0.127 ± 0.001	0.042 ± 0.003
DeepSurv	0.682 ± 0.001	0.969 ± 0.002	0.110 ± 0.003	0.030 ± 0.009	0.670 ± 0.006	0.996 ± 0.001	0.117 ± 0.001	0.018 ± 0.003
Nnet-Survival (Logistic Hazard)	0.668 ± 0.001	0.976 ± 0.001	0.131 ± 0.003	0.037 ± 0.002	0.642 ± 0.005	0.980 ± 0.001	0.110 ± 0.002	0.042 ± 0.002
Stage II								
Cox-Time	0.689 ± 0.003	0.988 ± 0.001	0.106 ± 0.001	0.007 ± 0.003	0.676 ± 0.006	0.978 ± 0.003	0.110 ± 0.002	0.011 ± 0.001
DeepHit	0.722 ± 0.001	0.988 ± 0.001	0.122 ± 0.001	0.080 ± 0.007	0.724 ± 0.003	0.842 ± 0.005	0.129 ± 0.002	0.001 ± 0.003
DeepSurv	0.672 ± 0.003	0.965 ± 0.003	0.105 ± 0.001	0.029 ± 0.001	0.663 ± 0.006	0.993 ± 0.001	0.104 ± 0.001	0.026 ± 0.002
Nnet-Survival (Logistic Hazard)	0.661 ± 0.001	0.977 ± 0.001	0.110 ± 0.001	0.038 ± 0.003	0.618 ± 0.002	0.984 ± 0.001	0.118 ± 0.001	0.024 ± 0.001
Stage III								
Cox-Time	0.642 ± 0.001	0.981 ± 0.002	0.091 ± 0.003	0.008 ± 0.001	0.709 ± 0.005	0.968 ± 0.004	0.080 ± 0.001	0.011 ± 0.001
DeepHit	0.621 ± 0.002	0.981 ± 0.001	0.094 ± 0.001	0.052 ± 0.003	0.703 ± 0.004	0.973 ± 0.001	0.085 ± 0.001	0.089 ± 0.007
DeepSurv	0.660 ± 0.004	0.984 ± 0.006	0.089 ± 0.003	0.024 ± 0.002	0.666 ± 0.014	0.993 ± 0.002	0.079 ± 0.002	0.024 ± 0.001
Nnet-Survival (Logistic Hazard)	0.627 ± 0.002	0.944 ± 0.009	0.091 ± 0.003	0.005 ± 0.002	0.604 ± 0.005	0.944 ± 0.003	0.087 ± 0.002	0.064 ± 0.007

## Data Availability

Original data were obtained from SEER-Medicare under a two-tiered review process. SEER-Medicare data are available to investigators upon review.
